# Nutritional interventions for late-life depression: evidence, mechanisms, and clinical assessment tools

**DOI:** 10.3389/fnut.2026.1789372

**Published:** 2026-06-04

**Authors:** Ze-Kun Li, Yao Gao, Han-Ni Li, Yi-Xuan Peng, Jiao-Jiao Qiao, Sha Liu, Xin Yan

**Affiliations:** 1 Drug Clinical Trial Institution, First Hospital of Shanxi Medical University, Taiyuan, China; 2 Nursing College, Shanxi Medical University, Taiyuan, China; 3 Department of Psychiatry, First Clinical Medical College/First Hospital of Shanxi Medical University, Taiyuan, China; 4 Shanxi Key Laboratory of Artificial Intelligence Assisted Diagnosis and Treatment for Mental Disorders, First Hospital of Shanxi Medical University, Taiyuan, China

**Keywords:** assessment tools, dietary patterns, gut-brain axis, late-life depression, neuroinflammation, nutritional intervention

## Abstract

Late-life depression (LLD) is common and disabling in older adults, and current pharmacological or invasive treatments are often limited by comorbidity and tolerability. Nutrition is a modifiable target with potential clinical value. This review examines the epidemiological association between nutritional status and LLD. It synthesizes evidence on essential nutrients and dietary patterns, discusses potential underlying mechanisms, and evaluates the clinical utility of nutritional assessment tools—such as the Geriatric Nutritional Risk Index (GNRI), the Mini Nutritional Assessment (MNA) combined with the Geriatric Depression Scale (GDS), and the Comprehensive Geriatric Assessment (CGA)—for identification and intervention. Overall, malnutrition and nutritional risk are consistently associated with greater depressive symptom burden in later life, and emerging data suggest that chrono-nutrition (particularly higher energy intake at breakfast) may be a relevant, under-recognized modifier of depression risk. Mechanistically, nutrition may influence LLD through neuroinflammation, neuroplasticity, gut–brain axis signaling, and oxidative/mitochondrial pathways. Pattern-based strategies appear most actionable: higher adherence to Mediterranean-type diets and Mediterranean–Dietary Approaches to Stop Hypertension Intervention for Neurodegenerative Delay (MIND) diets is generally linked to fewer depressive symptoms, whereas Western/processed-food patterns are generally associated with adverse outcomes; for plant-based approaches, dietary quality is critical. For nutrient-focused interventions, effects remain heterogeneous; benefits may depend on baseline deficiency, inflammatory status, and for omega-3, the eicosapentaenoic acid (EPA) to docosahexaenoic acid (DHA) ratio and dose. For clinical implementation, we highlight an assessment-to-intervention workflow integrating GNRI-based rapid stratification, MNA plus GDS screening, and CGA-guided multidisciplinary management for frail or complex patients. Future research should prioritize adequately powered randomized controlled trials (RCTs) with standardized protocols, biomarker-informed and genotype-aware stratification, and interdisciplinary translation to optimize nutrition-based prevention and comprehensive management of LLD.

## 1 Introduction

Late-life depression (LLD) commonly refers to clinically diagnosed depression or clinically meaningful depressive symptoms occurring in later life, typically among adults aged 60 years or above. Beyond the presence of low mood and decreased interest, the clinical manifestation of LLD often focuses on somatic symptoms, including sleep disturbances, fatigue, altered appetite, and pain, in addition to cognitive symptoms such as impaired attention and executive function (1–3). Approximately 20% of adults aged 60 and above experience psychiatric or neurological disorders (4). LLD is intricately linked with frailty, cardiovascular and cerebrovascular diseases, dyslipidaemia, dysglycaemia, chronic inflammatory states, and psychosocial stressors. The etiology and pathological pathways of LLD exhibit considerable heterogeneity (5). In contrast to younger adults, the impact of depression on physical functioning and cognitive abilities in older adults is more pronounced, potentially jeopardizing their capacity for independent living. Regrettably, mental health issues within this demographic are often erroneously attributed to the natural aging process, resulting in inadequate treatment. Thus, the identification and management of LLD is of paramount importance.

Current treatment strategies for LLD include antidepressant pharmacotherapy, psychotherapy, and physical interventions, all of which have demonstrated efficacy to varying degrees. However, their effectiveness in older adults is often constrained by substantial limitations. In older adults, age-related changes in drug metabolism and treatment response, combined with multimorbidity and polypharmacy, may increase the risk of adverse drug reactions, drug–drug interactions, and poor treatment tolerance (6). Common antidepressant-related side effects—such as anticholinergic burden, cardiovascular instability, and metabolic disturbances—may further compromise adherence and safety in this population. Psychotherapeutic interventions, while beneficial, may be less accessible or less effective in individuals with cognitive impairment, sensory deficits, or reduced mobility. Collectively, these challenges contribute to variable treatment response, poor long-term adherence, and a high risk of relapse, underscoring the need for complementary and more sustainable management approaches. In this context, nutrition—an essential and modifiable lifestyle factor—has emerged as a promising component of holistic care for geriatric depression (7). Older adults are particularly vulnerable to insufficient protein intake, micronutrient deficiencies, and decreased gut microbiota diversity. Additionally, ageing-related immune dysregulation, oxidative stress, and impaired vascular endothelial function create a conducive environment for the onset of depression (8, 9). Recent research further substantiates that malnutrition or nutritional risk in older adults significantly heightens the incidence of depression (10). Nutritional supplementation, acknowledged for its effectiveness and safety, is commonly utilized in clinical settings to alleviate depressive symptoms. However, it is important to recognize that while the influence of nutrition on depression in the general population is extensively researched, nutritional interventions in older adults encounter challenges, including side effects, comorbidities, and other obstacles.

The long-term impact of nutrition on geriatric depression is unclear, necessitating further research. This review synthesizes current evidence and propose research prospects from five aspects, including nutritional status, nutritional components, dietary patterns, potential mechanisms and clinical assessment tools.

## 2 Epidemiological links between nutritional status and late-life depression

A notable relationship exists between malnutrition and the incidence of depression among elderly populations, rendering this association a crucial issue in geriatric health. For instance, a particular study revealed that around 81.5% of older participants exhibited varying levels of depressive symptoms, with 20.4% classified as malnourished (11). Elderly individuals suffering from depression showed markedly lower daily intakes of energy, protein, fat, zinc, vitamin B12, and vitamin D in comparison to their non-depressed counterparts, with deficiencies in these nutrients being linked to the onset of depression (12). Moreover, various epidemiological investigations substantiate this connection. A prospective study involving 105 hospitalized older patients diagnosed with depression demonstrated the concurrent presence of malnutrition and depression, with the risk of malnutrition positively correlating with the severity of depressive symptoms (13). Additionally, a cross-sectional analysis conducted in a nursing home in Mexico City found that 59.9% of participants were at risk of malnutrition. Furthermore, 27.9% displayed mild depressive symptoms, while 11.4% were diagnosed with major depression. Those experiencing depressive symptoms were approximately five times more likely to be malnourished compared to non-depressed individuals (11). Similar research conducted among elderly populations in other countries has revealed similar trends (14). Taken together, these studies illustrate a significant link between malnutrition, nutritional risk, and depressive symptoms in older adults.

However, the interpretation of current epidemiological evidence requires caution. The strength and interpretation of the observed associations may partly reflect cross-cultural differences in habitual diet, food availability, meal structure, and socially shaped eating behaviors (15, 16). In addition, methodological heterogeneity remains substantial: depression has been identified using screening scales, self-reported symptoms, or clinical diagnosis, whereas nutritional status has been assessed using screening tools, biomarkers, anthropometric indices, dietary recall, or dietary pattern scores. These differences in exposure definition and outcome measurement limit direct comparability across studies and should be considered when assessing the clinical applicability of the literature (11–13, 17, 18) Representative studies and their measurement characteristics are summarized in Supplementary Table 1.

Equally important to consider is the timing of meals alongside nutritional content. The core of chrononutrition, which addresses “when to eat,” is increasingly acknowledged for its influence on the risk of depression. Evidence suggests that aligning energy intake with circadian rhythms can mitigate depression risk. For example, a greater energy intake during breakfast is associated with a reduced risk of depression (19). An optimal distribution of energy intake throughout the day, particularly with a high-energy breakfast, may help reset the oscillations of circadian clock genes and alleviate disruptions in circadian rhythms. Such disruptions are linked to altered expression of clock genes, abnormalities in cortisol levels, inflammation, and imbalances in gut microbiota, all of which can elevate the risk of depression. In contrast, a low energy intake at breakfast significantly heightens the risk of depression (20). These findings suggest that, beyond nutritional content or “what to eat,” meal timing or “when to eat” may also be a relevant but under-recognized factor influencing depression risk in older adults.

In addition to nutritional status in later life, early-life and adolescent malnutrition may also contribute to the development of LLD from a life-course perspective. Nutritional deprivation during critical developmental periods may have long-term effects on brain maturation, stress-response systems, immune regulation, metabolic health, and neuroplasticity, which may increase vulnerability to depressive symptoms in later life (21). Evidence from early-life undernutrition and famine-exposure studies suggests that malnutrition during fetal life, childhood, or adolescence may be associated with a higher risk of depressive symptoms in adulthood and old age (22). However, these findings should be interpreted cautiously, as early nutritional deprivation is often accompanied by socioeconomic adversity, infection, trauma, and other environmental stressors. Future studies on LLD should therefore consider not only current nutritional status, but also life-course nutritional history, including childhood food insecurity and adolescent malnutrition.

In conclusion, current epidemiological findings support a robust relationship between poor nutritional status and depressive symptoms in older adults, while also highlighting the importance of cultural context, methodological consistency, meal timing, and life-course nutritional exposures in interpretation. These observations provide a rationale for incorporating routine nutritional assessment into the clinical management of older adults at risk of depression and for further investigating nutrition-related strategies for prevention and early intervention.

## 3 The influence of nutrition on late-life depression

### 3.1 The role of nutritional components

#### 3.1.1 Vitamins

B vitamins, including vitamins B6, B9, and B12, as well as vitamin D, have been widely implicated in late-life depression. Current evidence suggests that these vitamins may be relevant to LLD through pathways related to neurotransmitter balance, inflammation, and neuroplasticity, although the clinical effects of supplementation remain heterogeneous (23). A comprehensive longitudinal investigation involving 3,849 participants indicated that older individuals manifesting new depressive symptoms had markedly lower initial levels of vitamin B12 and folate, with a deficiency in B12 correlating with a 51% increase in the risk of depression over 4 years (24) (Table 1). While randomized controlled trials have substantiated the notion that supplementation with folate and B12 can enhance cognitive performance in older adults suffering from depression (25), the efficacy of these vitamins as antidepressants remains a topic of debate. For example, a 2-year intervention conducted by De Koning reported no significant outcomes (26), hinting that variables such as dosage, treatment duration, and initial nutritional status could significantly impact therapeutic efficacy.

**TABLE 1:** Summary of Nutritional and dietary pattern interventions for LLD: mechanisms, clinical effects, limitations, and practical recommendations.

Intervention category	Specific intervention strategy	Putative mechanisms	Main outcomes	Limitations	Practical clinical recommendations	Target population	References
Vitamins	Supplementation	Hcy↓; neurotransmitter synthesis; modulates neuroinflammation and immune responses; inhibition of NF-κB and the NLRP3 inflammasome.	Deficiencies in vitamin B12 and vitamin D are linked to higher depression risk; B12 supplementation may improve cognition.	Effects vary by dose and baseline nutritional status.	Assess status first; supplement if deficiency or risk is present; individualize dose	Suspected deficiency, limited sun exposure, poor intake, bone health risk, or elevated homocysteine	(24, 27, 28)
Unsaturated fatty acids / omega-3 fatty acids	Supplementation 1-2 g/day	Anti-inflammatory effects; improved membrane fluidity; modulation of brain-derived neurotrophic factor (BDNF).	Greater benefit observed in subgroups with elevated inflammation.	EPA:DHA ratio and dose appear critical.	Prioritize high-EPA formulations; monitor the EPA:DHA ratio, dose, and treatment duration; consider anti-inflammatory diet	Low fish intake, possible inflammation-related depression, or recurrent symptoms	(32, 36)
Protein / tryptophan	Dietary intake ≥ 1–1.5 g/kg/day	Tryptophan as a 5-HT precursor; dysregulation of the tryptophan–kynurenine pathway.	Lower tryptophan and higher kynurenine levels are associated with depression.	Tryptophan metabolic imbalance appears particularly prominent in LLD.	Ensure adequate protein intake; consider tryptophan only after intake is optimized	Poor intake, frailty, weight loss, sarcopenia risk, or malnutrition	(44)
Dietary fiber	Increased whole grains, legumes, vegetables, fruits	SCFAs (e.g., butyrate)↑; modulation of the gut–brain axis.	Higher fiber intake may reduce depressive symptoms.	Potential sex differences; possible reverse causality.	Increase gradually from whole foods; monitor gastrointestinal tolerance	Constipation, poor dietary diversity, or low fiber intake	(46)
Probiotics	Fermented foods or supplements	Restores gut microbiota balance; inflammation↓.	Slight improvement in depressive symptoms, but effects are not significant in older adult populations in some studies.	Dose and strain-specific effects.	Use probiotics cautiously and selectively; monitor gut tolerance and depressive symptoms	Suspected gut microbiota imbalance	(48, 49)
Mediterranean diet	High vegetables, fruits, whole grains, legumes, nuts, olive oil; moderate fish	Anti-inflammatory and antioxidant effects; higher omega-3 intake; gut microbiota modulation.	Higher adherence is associated with fewer depressive symptoms.	Regional and sex differences.	Promote gradual adoption; tailor to comorbidities and preferences	Low diet quality, cardiometabolic risk, chronic inflammation	(50, 51)
MIND diet	Mediterranean + DASH; leafy greens, berries, nuts, whole grains, fish, olive oil	Anti-inflammatory; neuroprotective effects.	Higher adherence is associated with fewer depressive symptoms.	More longitudinal evidence is needed.	Adapt to local diet; emphasize leafy greens or other dark produce and reduce sweets and ultra-processed foods	At risk of depression or cognitive decline	(56)
Western diet	High sugar/fat, processed foods; low fruits/vegetables	Inflammation↑ and oxidative stress; gut dysbiosis.	Higher intake of processed foods is associated with increased depression risk.	Reverse causality.	Promote gradual replacement of ultra-processed foods with minimally processed alternatives rather than abrupt restriction	High intake of processed foods, refined grains, sugary drinks	(60, 62)
Plant-based diet	Emphasize high-quality plant foods; limit refined/fried plant foods	Antioxidant effects; gut–brain axis modulation; regulation of inflammatory pathways.	High-quality plant-based diets may be beneficial for depression.	Diet quality is critical.	Focus on diet quality; monitor vitamin B12 and omega-3 adequacy when intake is restricted	Vegetarian or plant-forward preference	(64)
Ketogenic diet	<50 g carbs/day; high fat	Neuroprotective effects; inflammation ↓; modulation of gut microbiota.	Moderate improvements in depressive symptoms have been reported.	Primarily short-term evidence.	Use cautiously, with monitoring of weight, intake, tolerance, and frailty risk	Selected metabolically stable individuals	(69)
Time-restricted eating	≤8-h eating window	Circadian rhythm alignment; modulation of inflammatory pathways.	May improve mood and sleep quality.	Longer-duration RCTs are needed.	Encourage regular meal timing; avoid late eating; monitor nutrition and weight	Irregular meal timing, breakfast skipping, circadian disruption, or overweight/obesity	(71)

BDNF, brain-derived neurotrophic factor; DHA, docosahexaenoic acid; EPA, eicosapentaenoic acid; Hcy, homocysteine; 5-HT, serotonin; LLD, late-life depression; NF-κB, nuclear factor kappa B; NLRP3, NOD-like receptor family pyrin domain-containing 3; RCT, randomized controlled trial; SCFAs, short-chain fatty acids; MIND, Mediterranean-DASH Intervention for Neurodegenerative Delay.

Vitamin D has also been associated with depression-related pathways relevant to immune regulation and brain function, although its clinical benefits in LLD remain inconsistent across studies. The prevalence of vitamin D deficiency is notably higher among older adults, attributed to factors such as limited sun exposure, reduced skin synthesis, and declining renal function. Numerous cohort studies and systematic reviews have indicated a link between low vitamin D levels and an elevated risk of depression (27, 28). However, the antidepressant effects of vitamin D3 supplementation have not been conclusively validated in clinical trials and meta-analyses (29, 30), potentially due to limitations such as small sample sizes, insufficient dosages, or short intervention periods. Recent dose-response meta-analyses suggest a gradient effect of vitamin D3 on depressive symptoms, where a daily increase of 1000 international units (IU) results in a modest improvement in symptoms, peaking at 8000 IU/day, beyond which the additional benefits become minimal (31).

Other vitamins, particularly lipid-soluble vitamins, may also be relevant to LLD, although direct evidence remains limited. Vitamin A-related carotenoids have been associated with a lower risk of depressive symptoms in older adults, while vitamin E and vitamin K have also been discussed in relation to depressive symptoms and neurological function. However, current evidence for vitamins A, E, and K in LLD is mainly observational or mechanistic, and remains insufficient to support routine supplementation in the absence of confirmed deficiency (23). Therefore, these vitamins may be considered as part of broader nutritional assessment rather than as independent intervention targets for LLD.

Overall, B vitamins and vitamin D currently have the most direct evidence in relation to LLD: B vitamins may be more prominent in prevention, while vitamin D supplementation is recommended for individuals with deficiency or bone health risks. Evidence for other lipid-soluble vitamins remains emerging. Current evidence emphasizes the need for individualized treatment approaches. Future large-scale, long-term RCTs are required to clarify optimal treatment windows and dosages. This will help translate mechanistic potential into clinical practice.

#### 3.1.2 Unsaturated fatty acids

Unsaturated fatty acids constitute a beneficial class of fats for human health. Among these, omega-3 fatty acids—particularly eicosapentaenoic acid (EPA) and docosahexaenoic acid (DHA)—have received considerable attention in the prevention and management of LLD, with potential relevance to inflammatory regulation and neural function (32). Observational studies indicate that high omega-3 intake or blood omega-3 levels correlate with reduced depression risk (33). However, in Japanese elderly populations, where long-term high fish consumption leads to elevated baseline omega-3 polyunsaturated fatty acid levels, conventional supplementation showed no additional benefit, suggesting potential advantages may be concentrated in those with insufficient intake (34). Results from randomized controlled trials also exhibit heterogeneity. In elderly individuals with mild cognitive impairment, both the EPA group (1.67 g EPA + 0.16 g DHA/day) and the DHA group (1.55 g DHA + 0.40 g EPA/day) demonstrated reductions in Geriatric Depression Scale (GDS) scores (35). In elderly women, daily supplementation with 2.5 g omega-3 (1.67 g EPA + 0.83 g DHA) significantly alleviated depression (36). However, for patients with stable mood in late-life depression (LLD), 1.2 g EPA + 1 g DHA daily only reduced relapse risk without markedly improving depressive or anxiety symptoms or inflammatory markers, suggesting a primarily preventive effect (37). Systematic reviews and meta-analyses further indicate (38, 39) that overall, omega-3 fatty acids yield modest improvements or help maintain stable conditions in geriatric depression, influenced by dosage, baseline depression severity, intervention duration, and EPA to DHA ratio. Although these findings are inconsistent, existing evidence favors high-EPA supplements with an EPA to DHA ratio of 2:1 or greater for confirmed depression, particularly when inflammation is present. Clinical guidelines recommend an initial dose of ≥ 1 g net EPA, escalating to ≥ 2 g/day post-tolerance to enhance efficacy.

Overall, omega-3 fatty acids may contribute to the management of LLD, although the available evidence remains heterogeneous and their effects appear to depend on dosage, the EPA:DHA ratio, baseline inflammatory status, and treatment duration.

#### 3.1.3 Protein

Protein may influence LLD partly through its role as a source of essential amino acids, particularly tryptophan. As the precursor of serotonin, inadequate tryptophan availability may be relevant to mood dysregulation in older adults. Observational studies suggest that older adults with mild to moderate depression tend to have lower tryptophan intake and altered kynurenine-related metabolites, and abnormalities in tryptophan metabolism appear to be more prominent in LLD, especially in those with cognitive impairment (40–42). However, high-quality randomized controlled trials specifically evaluating protein supplementation for geriatric depression remain limited. From a clinical nutrition perspective, the most practical implication is to ensure adequate protein intake. The PROT-AGE group recommends at least 1.0–1.2 g/kg/day for adults aged 65 years and older, increasing to 1.2–1.5 g/kg/day in those with acute or chronic illness or at risk of malnutrition (43). In relation to population-based evidence for depression, research utilizing National Health and Nutrition Examination Survey (NHANES) data indicates an approximately L-shaped relationship between total protein intake and the risk of depression: individuals with lower intake levels exhibit a higher risk, while the marginal benefits of increased intake diminish beyond a certain threshold (44).

Overall, protein should be considered a foundational nutritional component in the management of LLD. In clinical practice, the priority is to ensure adequate protein intake according to guideline-recommended thresholds, while the mechanistic relevance of tryptophan metabolism is discussed in section “4. Mechanisms.”

#### 3.1.4 Other nutritional components

Dietary fiber, an essential element of healthy diets, is associated with reduced depression risk, though gender differences exist: as evidenced by the correlation between higher fiber consumption and reduced depressive symptoms in women, a relationship not observed in men. This difference may be attributed to varying dietary patterns; for instance, women’s increased intake of sugar-rich fruits, could obscure the connection, particularly given the established association between high fructose consumption and behaviors indicative of depression (45). Additionally, the possibility of reverse causation must be acknowledged; individuals experiencing depression may modify their dietary habits, potentially leading to a preference for ultra-processed foods and consequently a decrease in fiber intake (46). Dietary fiber may represent a promising intervention for LLD, potentially through microbiota-related pathways. However, further focused studies within elderly populations are necessary to confirm causality.

Probiotics, which are live microorganisms found in fermented foods like yogurt, contribute to the maintenance of gut microbiota equilibrium. Evidence suggests that the intake of fermented foods may positively impact mood (47). Clinical supplementation with probiotics has the potential to lower the incidence of depression and mitigate symptoms such as low mood or anhedonia, although the majority of evidence available is cross-sectional (48). A randomized controlled trial shows (49) that the efficacy of probiotics in mitigating depression is limited among individuals aged over 65, with more significant benefits reported in those under 60. This variation may be related to distinct characteristics of gut microbiota in older adults, alterations in immune responses, and the specific strains and dosages used, highlighting the necessity for precise intervention designs tailored for this demographic.

In clinical settings, a more practical approach is to encourage the consumption of whole grains, legumes, vegetables, fruits, and moderate amounts of fermented dairy products, provided that digestive tolerance is ensured.

### 3.2 The influence of dietary patterns

#### 3.2.1 The Mediterranean diet

The Mediterranean diet emphasizes vegetables, fruits, whole grains, legumes, nuts, and olive oil, with moderate fish and fermented dairy. Owing to its high content of fiber, unsaturated fats, and antioxidant-rich foods, it has been considered a potentially beneficial dietary pattern for the prevention and management of LLD. Recent studies suggest that higher adherence is associated with fewer depressive symptoms in LLD. Greater intake of fish, olive oil, vegetables, and fruits correlates with lower symptom severity, whereas higher consumption of sugar-sweetened beverages and red meat is linked to worse symptoms (50). Tea intake and physical activity have also been reported in relation to depression outcomes, highlighting potential lifestyle clustering and confounding. Evidence is heterogeneous (51). An Italian high-risk cohort suggested stronger benefits in women with high adherence (52), supported by some studies (16). However, cross-sectional studies in Australian community-dwelling older adults and in individuals aged ≥ 75 years in Galicia, Spain found no significant association with depression assessed by the GDS (53), possibly due to differences in design, population context, dietary assessment, and residual confounding. Cultural dietary background may be another important source of heterogeneity. In non-Mediterranean settings, adherence scores may not fully capture the intended nutrient profile of the Mediterranean diet, because the availability, culinary use, and cultural acceptability of core components such as olive oil, seafood, legumes, and fermented dairy vary substantially. As a result, nominal adherence to a Mediterranean-style pattern may reflect different food combinations across populations, potentially contributing to inconsistent associations with depressive symptoms in older adults (54).

Overall, the literature supports a potential preventive and/or symptom-ameliorating role of the Mediterranean diet in older adults, with more pronounced effects among those with high adherence and possibly among women. Clinically, efforts should prioritize improving adherence to the dietary pattern: increasing plant-based foods and fish, using (extra virgin) olive oil as the main culinary fat, and limiting sugar-sweetened beverages and red meat. Further longitudinal studies and randomized controlled trials are needed to strengthen causal inference and clarify modification by sex, comorbidity, and lifestyle factors.

#### 3.2.2 MIND diet

The Mediterranean–Dietary Approaches to Stop Hypertension Intervention for Neurodegenerative Delay (MIND) diet integrates key elements of the Mediterranean diet and the Dietary Approaches to Stop Hypertension (DASH) dietary pattern, with a specific focus on brain health. It emphasizes higher intakes of leafy greens, berries, nuts/legumes, whole grains, fish, and olive oil while limiting saturated fat and highly processed, sugar-rich foods (55). Observational cohort evidence suggests that higher DASH and MIND scores are associated with a lower incidence of depressive symptoms in older adults over time, and these associations remain robust after adjustment for major confounders including age, energy intake, education, sex, antidepressant use, cardiovascular disease, and physical activity (56). For clinical translation, a pattern-based approach may be practical: increasing daily leafy greens, regularly consuming berries, nuts/legumes and fish, substituting refined staples with whole grains, and reducing red meat, desserts, and sugar-sweetened beverages. Cultural adaptation may improve feasibility. In Chinese older adults, a culturally adapted cMIND pattern has similarly been associated with reduced incidence of depressive and anxiety symptoms, supporting local substitutions such as blending olive oil with rapeseed or tea-seed oil and replacing berries with seasonal dark-colored fruits (57). This also suggests that the mental health effects of dietary patterns may depend not only on the theoretical model of the diet itself, but also on how successfully it is culturally translated into locally accessible, affordable, and acceptable foods. Such adaptation is particularly important in older adults, whose long-established food preferences and household eating practices may strongly influence long-term adherence. To support adherence and minimize nutritional risk, family involvement and routine monitoring of weight, appetite, and fatigue may be considered, particularly in frail individuals. Some studies further suggest that the association between MIND adherence and depression risk may involve biological ageing-related processes, although these findings remain preliminary (58).

Overall, the MIND diet shows promise for cognitive health and is favorably associated with depression-related outcomes in older adults, but the evidence base is predominantly observational. Large, long-term randomized controlled trials in older populations, integrating biomarker and mechanistic assessments, are needed to clarify causality and to define its role in the primary prevention of late-life depression.

#### 3.2.3 Western diet

The Western dietary pattern is typically characterized by high sugar and fat (often saturated fat), low dietary fiber, and a high proportion of processed foods, with relatively low intakes of fruits, vegetables, whole grains, and legumes (59). Current evidence consistently links the Western diet to adverse depression-related outcomes. In the Whitehall II longitudinal study, higher dietary inflammatory potential assessed by the Dietary Inflammatory Index (DII) was associated with a substantially higher risk of depressive relapse in women, with a 66% increase per 1-standard-deviation increment in DII (60). A prospective analysis of the same cohort also suggested that a sustained increase in processed food intake was associated with a gradual rise in subsequent depression risk (61). Studies focusing on ultra-processed foods (UPF) further suggest that higher UPF intake among middle-aged and older adults is associated with greater risks of overall mental disorders, depression/anxiety, and suicidal ideation, with metabolic factors partially mediating these associations; lifelong longitudinal data also indicate positive associations between UPF intake and subsequent depressive symptoms in community-dwelling older adults (62). Evidence from Asian populations is concordant: a Taiwanese cohort identified a Western-style pattern (higher meat/egg intake and lower fish/legume/vegetable/fruit intake) that was associated with increased risk of depressive symptoms over 8 years (63).

From a clinical perspective, gradual substitution may be more feasible than complete avoidance: replacing sugar-sweetened beverages with water or unsweetened tea; swapping part of refined grains for whole grains; substituting processed meats with fish, poultry, or soy-based options; choosing fruit or plain nuts over sugary snacks; and reducing fried takeaway foods in favor of steamed or stewed preparations.

#### 3.2.4 Plant-based diets

The relationship between plant-based dietary patterns and depression in older adults appears to depend more on dietary quality than on whether an individual is vegetarian. Accordingly, recent studies have increasingly used standardized plant-based dietary indices, including the overall Plant-Based Diet Index (PDI), the healthy Plant-Based Diet Index (hPDI), and the unhealthy Plant-Based Diet Index (uPDI), to quantify plant-based dietary patterns (64). Among these indices, the evidence is most consistent for the uPDI: lower-quality plant-based diets characterized by higher intakes of refined grains, sugar-rich foods, and fried plant-based items are associated with a greater burden of depressive symptoms and/or a higher risk of depression (65). In contrast, findings for PDI and hPDI are heterogeneous, with inverse, null, or even positive associations reported across different cohorts and analytical models, underscoring the importance of stratifying plant-based foods by quality (66). Such discrepancies may be attributable to differences in population structure (age, sex, comorbidities), cultural dietary context, dietary assessment methods, and residual confounding. Importantly, the cultural meaning of a “plant-based diet” also differs across settings: in some populations it may emphasize legumes, vegetables, and minimally processed staples, whereas in others it may include a higher proportion of refined carbohydrates, fried foods, or sugary plant-derived products. This variability may partly explain why plant-based dietary indices show inconsistent associations with depression-related outcomes across studies. Reverse causality should also be considered, as depressive states may prompt individuals to adopt self-perceived healthier vegetarian diets or to deliberately reduce animal-product consumption due to empathy for animal suffering, thereby influencing observed associations (67).

From a clinical perspective, a “quality-first” approach is recommended: prioritizing whole, minimally processed plant foods such as whole grains, vegetables, fruits, legumes, and nuts, while limiting refined carbohydrates, sugary foods, and fried items. For individuals adhering to highly restrictive patterns (e.g., vegan diets), attention should be paid to nutrients relevant to neuropsychological function, with assessment and supplementation of vitamin B12 and long-chain omega-3 fatty acids considered when appropriate. Future large-scale prospective studies integrating dietary quality stratification with biomarker-based assessment of nutritional status are needed to clarify directionality and to identify subgroups most likely to benefit.

#### 3.2.5 Other diets

The ketogenic diet (KD) and time-restricted eating (TRE) have been proposed as novel dietary approaches for mental health, but their application in older adults should prioritize safety and feasibility. KD typically restricts carbohydrates to < 50 g/day while increasing fat intake. Preclinical work suggests potential effects on rewards-related pathways, stress biomarkers (e.g., cortisol), and the gut microbiome (68). Randomized trials in treatment-resistant depression report reductions in depression and anxiety severity with improved quality of life and functioning (69); however, direct evidence in LLD is lacking, and age-related metabolic and nutritional vulnerability necessitates cautious estimate with monitoring. TRE is commonly defined as limiting daily intake to an eating window of ≤ 8 h (70). Evidence remains mixed: cross-sectional studies in general adult populations have often found no clear associations between TRE and stress, depressive symptoms, or sleep, whereas age-stratified analyses suggest that adults aged ≥ 70 years who follow an approximately 8-h eating window may have a lower likelihood of psychological distress after adjustment for diet quality. Notably, this association attenuates after further adjustment for breakfast timing, implicating the temporal structure of intake—particularly breakfast timing—as a potential driver (71). Other studies indicate that mood-related benefits may require concurrent exercise (72), and in overweight/obese or type 2 diabetes populations, TRE appears to mainly influence weight outcomes without consistent mood improvement (73, 74).

Overall, KD and TRE are better positioned as research avenues or cautious, individualized trials in selected patients rather than universal strategies for LLD. In frail or nutritionally at-risk older adults, overly restrictive regimens may increase the risk of inadequate energy and protein intake, potentially worsening fatigue and mood. Where TRE is considered, a conservative approach (regular breakfast and dinner timing, avoidance of late-night snacking) with clinical supervision, monitoring of weight and nutritional intake, and consideration of exercise co-intervention may improve feasibility and reduce risk.

## 4 Mechanisms

**FIGURE 1:**
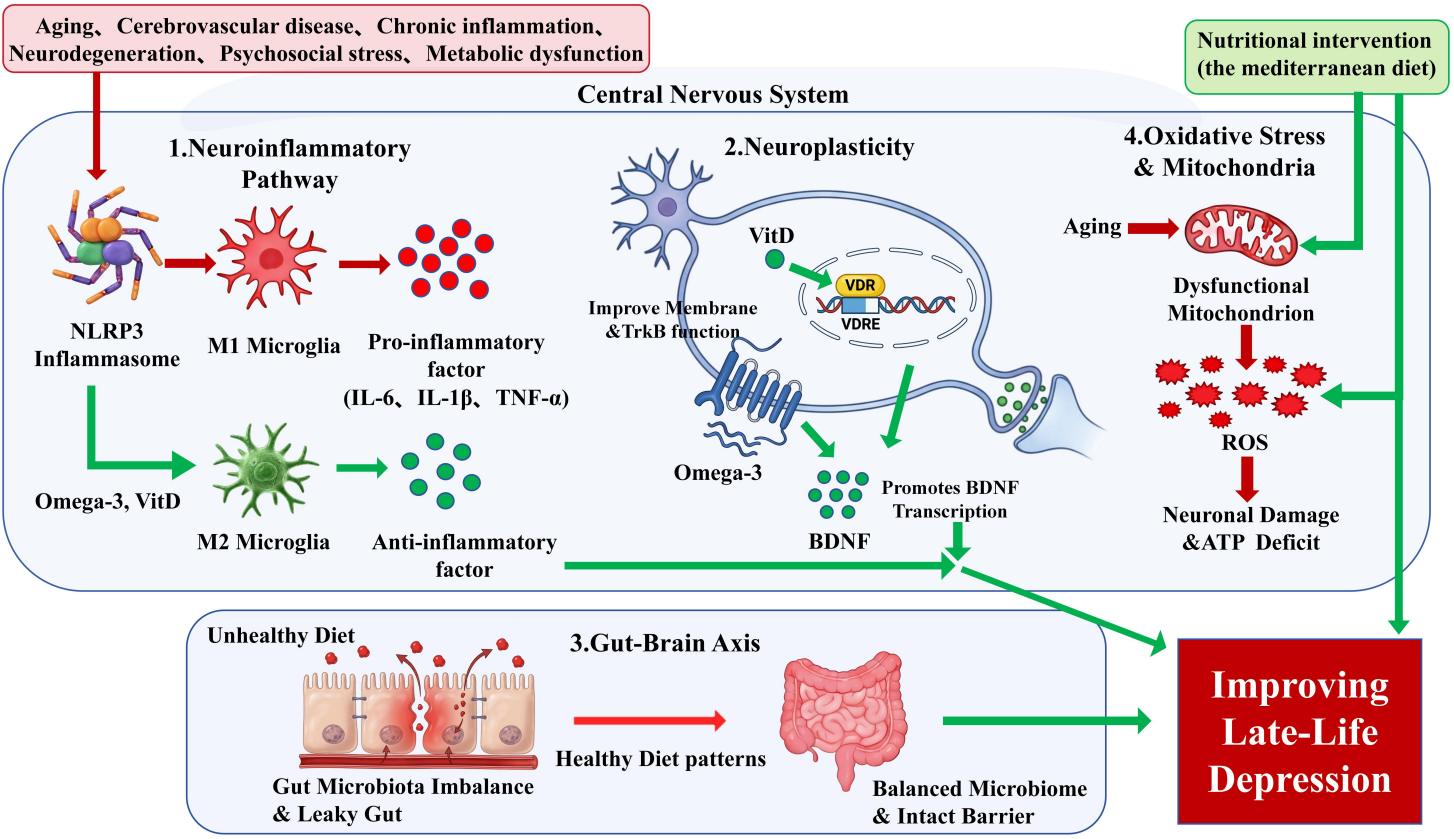
Mechanisms linking nutrition to late-life depression (LLD). NLRP3, NOD-like receptor family pyrin domain containing 3; IL-6, interleukin-6; IL-1β, interleukin-1 beta; TNF-α, tumor necrosis factor-alpha; VitD, vitamin D; TrkB, tropomyosin receptor kinase B; VDR, vitamin D receptor; VDRE, vitamin D response element; BDNF, brain-derived neurotrophic factor; ROS, reactive oxygen species; ATP, adenosine triphosphate; M1 microglia, pro-inflammatory microglial phenotype; M2 microglia, anti-inflammatory microglial phenotype. This figure summarizes the major underlying contributors to LLD, including aging-related, cerebrovascular, neurodegenerative, inflammatory, metabolic, psychosocial, and circadian factors. These contributors may interact with nutrition-related factors and converge on four key pathological pathways: neuroinflammation, impaired neuroplasticity, gut–brain axis dysregulation, and oxidative stress/mitochondrial dysfunction. Healthy dietary patterns and selected nutrients, such as omega-3 fatty acids, vitamins, dietary fiber, adequate protein/tryptophan, probiotics or fermented foods, and regular meal timing, may modulate these pathways, whereas Western or ultra-processed dietary patterns may aggravate inflammatory, metabolic, microbial, and oxidative disturbances.

Compared with depression in young adults, the etiology and neurobiology of depression in older adults exhibit greater heterogeneity, frequently intertwined with ageing processes such as cerebrovascular disease, neurodegenerative changes, and chronic low-grade inflammation (75). Inflammatory factors, stress-related neuroendocrine pathways, oxidative stress, mitochondrial dysfunction, and blood-brain barrier permeability all synergistically exacerbate depressive symptoms with advancing age. Nutrients influence mood and brain function through anti-inflammatory, neuroplasticity-promoting, gut microbiota-modulating, and antioxidant pathways, representing key modifiable targets discussed in this review (Figure 1).

### 4.1 Modulating neuroinflammation

Growing evidence suggests that chronic low-grade inflammation is involved in both the onset and progression of LLD, with inflammation-associated subtypes showing poorer clinical outcomes (76). The core mechanism of neuroinflammation involves chronic microglial activation: under stress, infection, or injury stimuli, microglia may adopt pro-inflammatory M1 or anti-inflammatory/reparative the M2 phenotypes. In LLD, microglia persistently favor the M1 state, releasing interleukin-1β (IL-1β), interleukin-6 (IL-6), and tumor necrosis factor-α (TNF-α). These inflammatory mediators may disrupt synaptic activity, reduce neurotrophic support, and shift tryptophan metabolism toward the kynurenine pathway, thereby contributing to depressive symptoms (77).

Consequently, inhibiting excessive M1 activation or promoting M2 phenotype represents a potential therapeutic strategy, particularly through nutritional interventions. For example, omega-3 polyunsaturated fatty acids suppress M1 while promoting M2 phenotype, concurrently inhibiting lipopolysaccharide-induced nuclear factor kappa B (NF-κB) signaling and reducing pro-inflammatory factor production (78). Vitamin D, via the vitamin D receptor (VDR), inhibits NF-κB and p38 mitogen-activated protein kinase while blocking NOD-like receptor family pyrin domain-containing 3 (NLRP3) inflammasome overactivation, thereby reducing IL-6, TNF-α, and IL-1β levels and upregulating interleukin-10 (IL-10) and transforming growth factor-β (TGF-β) (79). Suppression of microglial NF-κB signaling and NLRP3 inflammasome activation in the central nervous system can reduce pro-inflammatory mediator production. Meanwhile, M2 macrophages release IL-10 and TGF-β, which may promote neuroplasticity-related factors such as brain-derived neurotrophic factor (BDNF) and further inhibit NF-κB signaling, forming an anti-inflammatory regulatory loop. Therefore, inflammatory processes may represent a useful indicator of LLD risk and a modifiable target for nutrition-based intervention.

### 4.2 Enhancing neural plasticity

Adult neurogenesis in the hippocampal dentate gyrus and synaptic plasticity are crucial for mood and cognition, which are frequently impaired in LLD (80). BDNF is a key neurotrophic factor; peripheral and intracerebral BDNF levels are commonly reduced during depression but increase following successful treatment. Both omega-3 fatty acids and vitamin D enhance the BDNF/tropomyosin receptor kinase B (TrkB) pathway. Omega-3 and its metabolites modulate BDNF-related transcription and mitigate pro-inflammatory factor suppression of BDNF via anti-inflammatory effects; they also improve the lipid membrane microenvironment to enhance TrkB receptor responsiveness to BDNF (81). Vitamin D acts more directly on BDNF transcription: the BDNF promoter region contains vitamin D response elements (VDREs), and VDR binding promotes BDNF gene transcription (82). In addition, B vitamins, particularly folate and vitamin B12, may support neuroplasticity-related processes through methylation and homocysteine regulation, whereas adequate protein intake provides amino acid substrates, including tryptophan, for neurotransmitter-related pathways closely linked to serotonin and kynurenine metabolism (23, 40–42). Together, these nutritional factors may influence neural plasticity at complementary levels, including neurotrophic signaling, methylation status, neurotransmitter availability, and inflammatory regulation.

### 4.3 Modulation of the gut–brain axis

The gut microbiota and central nervous system form a bidirectional communication network that primarily influences mood through three pathways: ① The neurogenic pathway involves Bifidobacteria and lactobacilli producing neuroactive metabolites that activate trace amine-associated receptor 1 (TAAR1) to promote serotonin release or generate gamma-aminobutyric acid (GABA) to inhibit excessive excitation. These signals are transmitted to the brain via the vagus nerve. ② The immunological pathway includes butyrate inhibiting microglia-driven inflammation and upregulating neurotrophic factors through G protein-coupled receptor 109A (GPR109A). Additionally, bile acid derivatives reduce hippocampal inflammation via the farnesoid X receptor (FXR). Dysbiosis shifts tryptophan metabolism toward the kynurenine pathway, producing neurotoxic substances such as quinolinic acid that exacerbate inflammation and excitotoxicity (83). ③ The endocrine pathway features short-chain fatty acids promoting glucagon-like peptide-1 (GLP-1) secretion, regulating energy homeostasis, and partially normalizing hypothalamic-pituitary-adrenal (HPA) axis function. They may also influence circadian rhythms. Circadian disruption impairs intestinal barrier function and short-chain fatty acid signaling by altering gut microbial activity, thereby increasing the risk of depression (47).

Diet is a key factor in shaping the microbiota: high-fiber diets, fermented foods, probiotics, and high-quality plant-based dietary patterns may promote microbial diversity and the production of protective metabolites such as butyrate and other short-chain fatty acids, whereas high-fat, high-sugar, and ultra-processed dietary patterns are more likely to cause dysbiosis. Through these microbiota-derived metabolites, dietary fiber may influence intestinal barrier integrity, immune activation, vagal signaling, and hypothalamic–pituitary–adrenal axis regulation, thereby linking dietary intake with depressive symptoms (47–49, 84).

### 4.4 Oxidative stress and mitochondrial function

Oxidative injury and mitochondrial impairment may interact with LLD in a mutually reinforcing cycle. Ageing is accompanied by a diminished antioxidant capacity, characterized by reduced levels of antioxidants such as superoxide dismutase (SOD) and glutathione, as well as mitochondrial degeneration. Excessive reactive oxygen species (ROS) can promote lipid peroxidation, disrupt protein function, and damage DNA. These oxidative injuries may further activate apoptotic pathways and are often accompanied by increased oxidative damage markers and depleted antioxidant capacity in peripheral and brain tissues (85). Mitochondria serve as a pivotal hub: impaired oxidative phosphorylation results in insufficient adenosine triphosphate (ATP) production, while disruption of the electron transport chain triggers excessive ROS generation and cascading amplification (86). ROS may also attack mitochondrial DNA, causing mutations and impairing respiratory chain protein synthesis. Additionally, an imbalance in mitochondrial dynamics—such as excessive fission and fragmentation—weakens synaptic energy supply (87). Crucially, ageing diminishes mitochondrial biogenesis capacity, while elevated glucocorticoids induced by chronic stress further suppress mitochondrial function and amplify oxidative damage.

Notably, dietary patterns and nutritional status may modulate oxidative stress and mitochondrial homeostasis at multiple levels, thereby contributing to LLD risk and progression. Diets high in saturated fat and refined sugars are linked to obesity, insulin resistance, and low-grade inflammation, which may enhance ROS production and accelerate mitochondrial decline. In contrast, dietary patterns such as the Mediterranean, MIND, and high-quality plant-based diets—rich in polyphenols, fiber, ω-3 fatty acids, vitamins, and other antioxidant-related nutrients—have been associated with lower oxidative damage and more favorable mitochondrial metabolic profiles, potentially by strengthening endogenous antioxidant defenses, improving mitochondrial membrane lipid composition and respiratory-chain efficiency, and attenuating neuroinflammation (88). B vitamins may further support mitochondrial and energy metabolism through their roles as cofactors in cellular metabolic reactions and homocysteine regulation, whereas Western or ultra-processed dietary patterns may aggravate oxidative stress through excess saturated fat, refined sugars, and diet-induced metabolic dysfunction (23, 59, 62).

## 5 Nutritional assessment tools and their clinical application

The Geriatric Nutritional Risk Index (GNRI), Mini Nutritional Assessment (MNA) combined with the GDS, and Comprehensive Geriatric Assessment (CGA) serve complementary roles in assessing nutrition-related risk in LLD. GNRI is rapid and objective (albumin, weight) for outpatient follow-up or early admission triage, but values can be distorted by inflammation or fluid shifts and it provides limited intake/functional detail. Mini Nutritional Assessment-Short Form (MNA-SF) paired with the 15-item Geriatric Depression Scale (GDS-15) works well for community or primary-care screening, linking nutrition risk to depressive symptoms and guiding dietary action; however, MNA includes neuropsychological items that may overlap with GDS, and both tools are less reliable with cognitive or sensory impairment. CGA is preferred for frail, multimorbid, recurrently hospitalized, or functionally declining patients, enabling multidisciplinary planning and follow-up, but it is time and resource-intensive (Table 2).

**TABLE 2:** Practical utility of GNRI, MNA-SF, GDS-15, and CGA Across Clinical Care Levels in LLD.

Tool	Core domain assessed	Clinical care level(s)	Main clinical utility in LLD	Advantages	Limitations
GNRI	Nutritional risk	Outpatient follow-up, admission triage, acute inpatient care, rehabilitation	Helps identify nutrition-related risk in older adults with LLD and supports decisions on early nutritional intervention and closer clinical monitoring.	Simple, objective, and quick to calculate	Less informative for psychosocial and functional dimensions
MNA-SF	Malnutrition screening	Community care, primary care, outpatient clinics, hospital admission screening, long-term care	Supports first-line screening for malnutrition risk in older adults with late-life depression, especially when appetite loss, weight loss, or reduced intake are suspected.	Brief, validated, feasible for routine use	A screening rather than diagnostic tool
GDS-15	Depressive symptom screening	Community screening, primary care, outpatient clinics, inpatient wards, long-term care	Enables rapid identification of depressive symptoms and can be used with nutritional screening to identify patients needing further psychiatric or multidisciplinary evaluation.	Short, easy to administer, repeatable	Not diagnostic; positive findings require formal clinical assessment
CGA	Multidimensional geriatric assessment	Specialist geriatric services, multidisciplinary inpatient care, rehabilitation, transitional care, complex community cases	Best suited for frail, multimorbid, or functionally impaired older adults with LLD; supports integrated care planning across nutritional, psychiatric, and functional domains.	Comprehensive, individualized, multidisciplinary	Resource-intensive and more time-consuming

GNRI, Geriatric Nutritional Risk Index; MNA-SF, Mini Nutritional Assessment-Short Form; GDS-15, 15-item Geriatric Depression Scale; GDS-30, 30-item Geriatric Depression Scale; CGA, Comprehensive Geriatric Assessment; LLD, late-life depression. These tools are complementary rather than interchangeable. Brief screening instruments (e.g., MNA-SF, GDS-15) are used for initial identification, whereas full versions (e.g., MNA, GDS-30) are applied for second-line assessment when clinically indicated. GNRI provides objective nutritional risk stratification, while CGA is reserved for complex, frail, or multimorbid older adults requiring multidisciplinary management.

### 5.1 Geriatric Nutritional Risk Index (GNRI)

The GNRI quantifies nutrition-related risk in older patients using objective indicators such as serum albumin, actual body weight, and ideal body weight (89). It is suitable for rapid stratification during outpatient follow-up and early hospitalization, indicating the risk of nutrition-related adverse outcomes. It also serves as a risk indicator for nutritional and psychological comorbidities. The GNRI risk categories were defined according to Bouillanne et al. as major risk (GNRI < 82), moderate risk (82 ≤ GNRI < 92), low risk (92 ≤ GNRI ≤ 98), and no risk (GNRI > 98) (89). In an NHANES-based analysis of 2,946 older adults, GNRI values were significantly lower among participants with depression than among those without depression. GNRI values were inversely associated with depression prevalence and showed a non-linear relationship, with an estimated threshold around 104.18; below this threshold, depression prevalence increased more markedly (17). Although this threshold is higher than the previously stated stratification levels, it may reflect a specific cutoff relevant to depression risk. Clinical application may follow the “collection-calculation-stratification-intervention” workflow, which involves the following steps:

(1) Concurrently obtain height, weight, and albumin levels;
(2) Calculate GNRI using a standardized ideal body weight formula;
(3) Stratify risk based on the thresholds;
(4) Trigger further nutritional assessment, intervention planning, and re-evaluation.

In summary, the GNRI provides a novel approach for exploring cross-disease mechanisms linking nutrition and mental health, as well as for facilitating early clinical intervention.

### 5.2 Mini Nutritional Assessment (MNA) combined with Geriatric Depression Scale (GDS)

The MNA is a validated instrument for detecting nutritional risk in older adults (90). When used together with the GDS, it can help identify individuals who may require further evaluation for depressive symptoms. Community-based studies have reported an inverse association between nutritional status and the severity of depressive symptoms in older adults: depressed patients exhibit reduced food intake, with declining nutritional scores accompanying rising depression scores (18). Clinicians may adopt a stepwise screening approach from short to comprehensive assessments conducted concurrently. First, use the MNA-SF (the short form of the MNA; 3–5 min) for rapid screening, where scores of 12–14 indicate normal nutrition and scores ≤ 11 suggest risk (91). Those at risk should proceed to the full MNA or nutritionist assessment and initiate dietary intervention. Additionally, complete the GDS-15 (5–7 min) screening on the same day, where a score of ≥ 5 indicates risk of depression (92), which should be followed by further clinical interview and diagnostic assessment (93). Note three key points: ① The MNA includes items on “neuropsychiatric problems,” which may overlap with the GDS; these items must be analyzed separately to determine the source of low scores. ② The GDS is a screening tool, not a diagnostic measure. ③ cognitive impairment and hearing or vision issues may affect its reliability. Where necessary, incorporate caregiver information and correct the assessment results using CGA.

### 5.3 Comprehensive Geriatric Assessment (CGA)

Comprehensive Geriatric Assessment integrates multidimensional evaluations of nutrition, cognition, and affect, highlighting the close association between nutritional status and depression. It emphasizes a closed-loop management approach of “assessment-planning-follow-up,” suitable for frail elderly individuals with multiple coexisting conditions, recurrent hospitalizations, or functional decline (94). Core CGA nutritional parameters (e.g., BMI, serum albumin, MNA score) correlate with geriatric depression risk. Ju et al.’s study (95) demonstrated a 22.6% reduction in depression risk per 1-point increase in MNA score (OR = 0.774); calf circumference and serum albumin levels also showed negative correlations with depression. Large-scale studies by Salis et al. (96) further confirmed significantly elevated depression incidence among malnourished patients (OR = 4.97), which also correlated with functional dependency and fall risk, underscoring the value of systematic interventions targeting nutritional status. In clinical practice, a tiered approach may be adopted (97). During hospitalization or the acute phase, rapid screening via Nutritional Risk Screening 2002 or MNA-SF should be employed. Positive cases should proceed to CGA supplemented with Subjective Global Assessment (SGA)/GNRI nutritional assessment alongside GDS mood screening. Team discussions formulate comprehensive prescriptions—including nutrition, exercise rehabilitation, depression management, medication, and social support—and set quantifiable targets such as weight/intake, scores, activities of daily living (ADL), and falls. It is important to note that CGA should avoid redundant use of overlapping assessment scales; modules should be selected based on specific issues. Indicators such as albumin and weight are susceptible to inflammation and fluid status, necessitating interpretation from multiple sources. The results should be translated by clinicians into interventions and followed by re-evaluation to demonstrate CGA’s clinical value.

In summary, nutritional screening tools (e.g., GNRI, MNA) remain pivotal for identifying nutritional risks in older adults. Based on assessment outcomes, a systematic strategy combining individualized supplementation, dietary adjustments, and multi-domain lifestyle interventions is essential to reduce the incidence and severity of depression in this population.

## 6 Conclusion

A significant and unequivocal association exists between nutritional status and depression in later life, with deficiencies in specific nutrients and dysregulation of related metabolic pathways representing key pathophysiological mechanisms underlying LLD. Although nutritional assessment and multifaceted interventions hold promise for the prevention and management of LLD, the efficacy of specific nutritional supplements remains controversial. Future research should prioritize three areas: (1) well-powered RCTs with standardized protocols to determine the clinical effectiveness of nutritional interventions for LLD, with consistent reporting of supplement type, dosage, intervention duration, and outcome measures, while also accounting for cultural and regional differences that may influence reproducibility and real-world applicability; (2) deeper investigation of the interaction between nutrition and individual genetic background, alongside the identification of relevant biomarkers to support personalized prevention and treatment; and (3) stronger interdisciplinary collaboration across nutrition, psychiatry, bioinformatics, and public health to elucidate biological mechanisms and facilitate translation into effective interventions for older adults.
