# Supplementary material for: Nutritional interventions for late-life depression: evidence, mechanisms, and clinical assessment tools
Source: Front Nutr. 2026 Jun 4;13:1789372. doi: 10.3389/fnut.2026.1789372 (PMC13275259; doi:10.3389/fnut.2026.1789372)
Supplement: Supplementary file 1 [file Table_1.docx]

# Supplementary Tables

Supplementary Table S1. Methodological and measurement characteristics of representative studies on nutrition and LLD

| **Study**  **(author, year)** | **Study design** | **Population characteristics** | **Depression assessment** | **Nutritional / dietary assessment** | **Assessment timing / frequency** | **Methodological notes** | **Reference** |
| --- | --- | --- | --- | --- | --- | --- | --- |
| Velazquez-Alva et al. (2020) | Cross-sectional study | Nursing home residents(aged ≥ 65 years),n = 262, | GDS(symptom screening scale) | MNA(nutritional status screening tool) | Single assessment | Single-time-point assessment limits temporal inference between depressive symptoms and nutritional status. | (11) |
| Lobato et al. (2021) | Prospective cohort study with 6-month follow-up | Older inpatients with major depressive disorder(aged ≥ 60 years),n = 105 | DSM-5 diagnosis(clinical diagnosis) | MNA and anthropometric measures(nutritional status screening and anthropometry) | Baseline and 6-month follow-up | Provides clinically diagnosed depression rather than symptom screening alone. | (13) |
| Laird et al. (2023) | 4-year longitudinal study | Community-dwelling older adults(aged ≥ 50 years),n = 3,849 | CES-D-20 and CES-D-8(symptom screening scales) | Serum vitamin B and folate concentrations(biomarker-based nutritional assessment) | Three waves: baseline (2009-2011), follow-up 1 (2011-2012), and follow-up 2 (2014-2015) | Combines longitudinal depression assessment with biomarker-based nutritional measures. | (24) |
| Vyas et al. (2023) | 2-year randomized controlled trial | Older adults at elevated risk for LLD(men aged ≥ 50 years and women aged ≥ 55 years),n = 720 | DSM-IV diagnosis(Clinical diagnosis) and PHQ-9(symptom screening scale） | Vitamin D3 and marine omega-3 supplementation (supplement intervention; no habitual dietary assessment) | Baseline and 2-year follow-up ; daily supplementation. | Large supplementation trial reporting null findings for depressive outcomes. | (30) |
| Cheng et al. (2025) | 52-week double-blind randomized controlled trial | Older adults with LLD(aged ≥ 60 years),n = 39 | DSM-5 diagnosis(clinical diagnosis) | n-3 PUFA supplementation (supplement intervention; no habitual dietary assessment) | Baseline and weeks 4, 8, 16, 24, 32, 40, and 52 | Intervention study with repeated longitudinal outcome assessment over 1 year. | (37) |
| Cherian et al. (2021) | Prospective cohort study | Community-dwelling older adults in the Chicago area( mean age 80.4 years), n = 709 | CES-D-10(symptom screening scale) | Modified Harvard semi-quantitative FFQ;DASH, MIND, Mediterranean, and Western diet scores (dietary intake and dietary pattern assessment) | Baseline and annual follow-up over a mean of 6.53 years | Highlights repeated assessment of both dietary patterns and depressive symptoms in a cohort setting. | (56) |
| Niu et al. (2025) | Observational study(CLHLS 2018) | Older Chinese adults(aged ≥ 65 years),n = 6,945 | CES-D-10(symptom screening scale) | cMIND score (dietary pattern assessment) | Single assessment | Large-sample study using a culturally adapted dietary score with sensitivity analyses. | (57) |
| Zhou et al. (2025) | Retrospective cohort study(CLHLS,2008-2018) | Older Chinese adults(aged ≥ 65 years),n = 1,666 | Five-item depressive symptom questionnaire(self-reported depressive symptoms) | Simplified FFQ ;PDI and uPDI(dietary intake and dietary pattern assessment) | Baseline and repeated assessments across 2008–2018 (≥4 waves) | Trajectory analysis performed using a group-based trajectory model. | (66) |

Abbreviations: GDS, Geriatric Depression Scale; MNA, Mini Nutritional Assessment; DSM, Diagnostic and Statistical Manual of Mental Disorders; CES-D, Center for Epidemiologic Studies Depression Scale; PHQ-9, Patient Health Questionnaire-9; FFQ, food frequency questionnaire; DASH, Dietary Approaches to Stop Hypertension; MIND, Mediterranean-DASH Intervention for Neurodegenerative Delay; cMIND, culturally adapted Mediterranean-DASH Intervention for Neurodegenerative Delay; PDI, plant-based diet index; uPDI, unhealthy plant-based diet index; PUFA, polyunsaturated fatty acid; CLHLS, Chinese Longitudinal Healthy Longevity Survey.
